# In-Silico Molecular Binding Prediction for Human Drug Targets Using Deep Neural Multi-Task Learning

**DOI:** 10.3390/genes10110906

**Published:** 2019-11-07

**Authors:** Kyoungyeul Lee, Dongsup Kim

**Affiliations:** Department of Bio and Brain Engineering, Korea Advanced Institute of Science and Technology, Daejeon KS015, Korea; ney@kaist.ac.kr

**Keywords:** in-silico bioactivity prediction, virtual screening, multi-task learning, deep learning

## Abstract

In in-silico prediction for molecular binding of human genomes, promising results have been demonstrated by deep neural multi-task learning due to its strength in training tasks with imbalanced data and its ability to avoid over-fitting. Although the interrelation between tasks is known to be important for successful multi-task learning, its adverse effect has been underestimated. In this study, we used molecular interaction data of human targets from ChEMBL to train and test various multi-task and single-task networks and examined the effectiveness of multi-task learning for different compositions of targets. Targets were clustered based on sequence similarity in their binding domains and various target sets from clusters were chosen. By comparing the performance of deep neural architectures for each target set, we found that similarity within a target set is highly important for reliable multi-task learning. For a diverse target set or overall human targets, the performance of multi-task learning was lower than single-task learning, but outperformed single-task for the target set containing similar targets. From this insight, we developed Multiple Partial Multi-Task learning, which is suitable for binding prediction for human drug targets.

## 1. Introduction

Discovering novel compounds that bind to human proteins for use as drugs is gaining increased interest in clinical research. As metabolism within the human body is controlled by the interaction between molecules, predicting and validating potential molecular binding is essential for novel drug development [1]. In fact, the interaction between ligands and receptors, or drugs and proteins, is a key factor for drug effectiveness. Identifying novel drug-like compounds for the target protein is considered to be the first step in drug discovery [2]. However, only around 10% of candidate drugs are approved after clinical trials because of a lack of effectiveness or unexpected off-target effects [3,4].

In this context, various in-silico-based approaches in pharmaceutical research have been proposed to overcome the low success rate of novel drugs. Computer aided drug design (CADD) assists in the retrieval of viable drugs from a large-scale compound database, thereby substantially reducing the time and cost for clinical approval [5]. Predicting molecular binding between the ligand and target enables a highly-efficient virtual screening for specific targets and early avoidance of drug toxicity [6,7]. Recently, advances in data science due to deep learning techniques and General-Purpose computing on Graphics Processing Units (GPGPU) have established a new era of large-scale virtual screening with high efficiency and reliability. Deep learning, which has been successful in many research fields, has outperformed other machine learning tasks in drug-target binding prediction [1,8,9]. A deep learning algorithm is suitable for dealing with large-scale data, such as a ligand-target binding database, because of its flexible and efficient architecture [2]. Deep learning uses multiple layers with non-linear activation functions for effective learning of complex relationships hidden within the data [10]. In addition, backpropagation of deep neural networks fosters the learning of a substantial amount of data in a fast and efficient manner [11].

In the early stage of a machine learning approach for drug-target binding prediction, Quantitative Structure-Activity Relationship (QSAR) tasks based on linear regression have had considerable success [9]. Subsequently, models based on Bayesian theorem such as Bayesian neural networks were developed to improve performance [12,13]. More recently, Random forests (RFs) [14], Support Vector Machines (SVMs) [15], and other popular machine learning approaches have displayed successful results. Deep Neural Network (DNN), a highly effective algorithm at image, speech, and natural language recognition [16,17,18], first revealed its strength in molecular binding prediction at the Merck Kaggle competition in 2012. The winning team at the QSAR competition had a performance of more than 15% over the baseline [9]. Following this achievement, Unterthiner et al. performed a large scale QSAR research using DNN across 1230 targets from the ChEMBL database [19,20]. The area under curve (AUC) of the Receiver Operating Characteristics (ROC) was averaged across targets as a standard performance, demonstrating that DNN outperformed conventional methods such as SVM or Similarity Ensemble Approach (SEA) [21]. In addition, Ramsundar et al. constituted drug-target classification DNN with almost 40 million measurements across 200 targets using multiple softmax classifiers attached after several common layers for all targets [2]. Recently, a study compared various machine learning methods for binding prediction across 1310 bioassays from ChEMBL through an unbiased validation [22]. They found that the simple Feed Forward Neural Network (FNN) could outperform not only conventional methods such as SVM or RF, but also the latest deep learning techniques such as Graph Convolution (GC) and Long Short-term Memory (LSTM) models. The results also showed that the FNN model can predict drug activities almost as accurately as an in-vitro assay.

Previous works applying DNN for QSAR binding prediction commonly insist that multi-task learning helps to improve prediction performance. Multi-task learning refers to an approach that simultaneously trains a neural network that is shared by multiple tasks with output neurons for individual tasks [9]. In contrast, single-task learning refers to the training of multiple neural networks for respective tasks without common layers. In deep learning-based molecular binding prediction, the task would mean the prediction of molecular binding activities for target or assay. Multi-task learning is widely perceived to gain better performance than single-task learning [2,23]. In the former, a common feature among different tasks can be extracted from the shared layers to help immature tasks with limited data to predict proper results and reduce overfitting [2]. Because learning of a task is leveraged by the knowledge that is gained from other tasks, this phenomenon is called “transfer of knowledge”. Multi-task learning is especially attractive in this subject because there is a rather prominent scarcity of data for particular targets and known interactions are highly biased to popular targets [14,20]. In addition, because many compounds from drug-target interaction data have multiple targets, multi-task learning is more promising [22].

Nevertheless, for the practical purpose of predicting drug-candidates for human drug targets, the effectiveness of multi-task learning requires more precise examination. Firstly, the exact deep learning architecture that is used for multi-task learning is quite different for each study and no fair comparison has been made among them. Secondly, the target sets that are used in previous studies do not focus on the large-scale human genome, which obscures whether the performance is still valid for general human drug targets. Among around 30,000 human genomes, an estimated 600–1500 genes are sorted as “drug targets” [24]. In this study, we checked the prediction performance of multi-task learning for 1067 potential human drug targets in the ChEMBL database and compared the results to that achieved with single-task learning. Lastly, multi-task learning for various target composition must be verified as its performance is highly dependent on inter-task correlation and data composition [22]. One of the weaknesses of multi-task learning is the lack of task-specific nonlinear learning, which is an obstacle to sufficiently learn the difference between tasks [23]. Therefore, multi-task learning with dissimilar human targets could result in even lower accuracy. Such an adverse effect should be carefully examined to derive the best practice for binding prediction for human drug targets.

In this study, we compared various types of multi-task learning methods from previous researches using a normalized validation method. According to previous studies, the output neurons for multi-task learning architecture, which are task-specific layers, can be constituted as a multi-label classifier [9,20,22] or multiple binary classifiers [2,23]. The former strategy is fast and easy to implement, but its issue of unknown data should be considered. The output of multi-label classifier always returns the prediction across all targets including those where the activities are unknown. Such unknown values need to be treated so that they do not influence the network. The latter is a strict way to implement target-specific layer and data mini-batch can be generated independently for each target. However, it is relatively difficult to implement and costs larger computational time and memory for a substantial number of tasks. We built many different architectures for multi-task learning and compared them to single-task learning for various human drug targets. Also, the human targets were clustered based on sequence similarity at their binding domains through which we found that target similarity is highly important for the retrieval of reliable prediction by multi-task learning. To avoid any unexpected bias from compound specificity [25] and hyperparameter selection, the validation process was performed using nested cluster cross-validation [22,26]. Using the unbiased comparison method, we found that single-task learning and various multi-task learning had both strengths and weaknesses, depicting different performances based on target composition. Finally, we developed a novel strategy for molecular binding prediction of human drug targets, which performs multi-task learning for distinct target clusters multiple times.

## 2. Materials and Methods

### 2.1. Data Curation from the ChEMBL Database

The ChEMBL [19,27] database is a well-known source of large-scale biological activities that are frequently used in similar researches [20,22]. We used version 23 of ChEMBL which contains 11,538 targets and 1,735,442 compounds with 14,675,320 activities. One of the advantages of using the ChEMBL database is that it is provided as an MySQL [28], which allows researchers to efficiently curate large volumes of data using scripts. The scripts that were used to curate bioactivity data are presented in Supplementary File S-1. We curated targets that are denoted as single proteins and activities with confidence scores above 8 to ensure that binding activities were gathered from direct interactions between single proteins and compounds, not including protein complexes. Activities with binding affinities stronger than 10 μM were collected as active data, while activities with lower binding affinities were considered inactive data. Compound structures were extracted in Simplified Molecular Input Line Entry System (SMILES) format [29]. Species of targets were not considered at this stage. Instead, the taxonomy of targets was curated from the database. Moreover, amino-acid sequences of known binding sites of targets were curated for target clustering.

### 2.2. Data Representation of Compounds

The SMILES format of compound structures is a text-based representation of molecules and does not represent scalable features that are required for training. Thus, further modification for compound data must be facilitated for machine learning. First, the ChemAxon standardizer [30] standardized the SMILES formatted data using options “Neutralize”, “Clean 2D”, “Remove Fragment”, “Remove Explicit Hydrogens”, “Mesomerize”, and “Tautomerize”. Then, standardized SMILES data were transformed into feature vectors of structural fingerprints. In this study, we chose semi-sparse features for compound representation following the best practices that are available for binding prediction for assays in ChEMBL [22]. In addition, the results using Extended Connectivity Fingerprints (ECFPs) as a representation [31] were measured to verify whether our finding was consistent for various fingerprints. Changing the radius of the circle increment for the substructure, we validated both ECFP4 (diameter = 4 Å) and ECFP6 (diameter = 6 Å). An open-source software RDKit package [32] was used to retrieve the fingerprints.

### 2.3. Deep Neural Network Architectures

**Figure 1:**
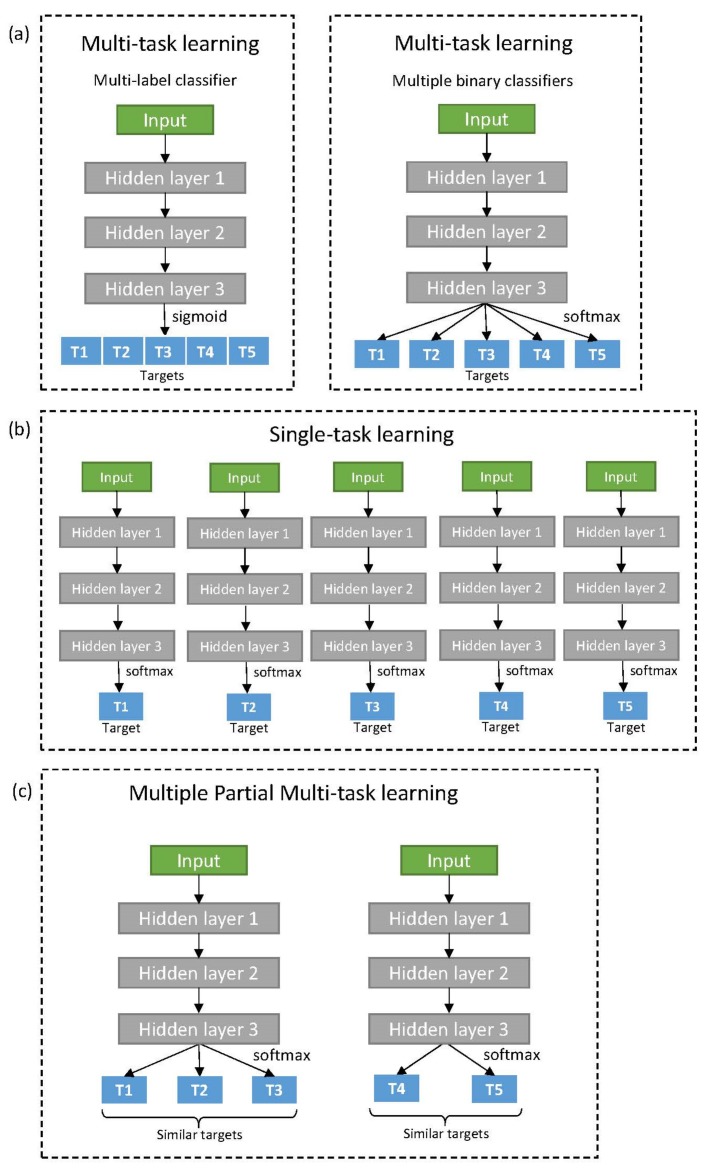
Various deep neural architectures for multi-task and single-task learning: (**a**) Multi-task learning architectures for two types of target specific output neuron; Multi-label classifier (left) and Multiple binary classifiers (right); (**b**) Single-task learning; (**c**) Multiple Partial Multi-task learning which separates targets into multiple partial target sets for training together by multi-task learning.

As mentioned previously, multi-task architecture in deep learning can adapt a multi-label classifier or multiple binary classifiers as its task-specific output layer (Figure 1a). Because of structural simplicity and comparable performance, the multi-task architecture using a multi-label classifier has been widely used [9,20,22]. However, a multi-label classifier always returns a prediction across all targets, even for targets with unknown activity with the input compound. Normally, such unknown data is processed to not be trained by backpropagation (masking). During mini-batch generation, the balance between prediction tasks tends to be skewed to the major targets with many activities. Some of the previous studies compensate for such effect using task-specific weights when defining loss function (task-weighting) [20]. We built two multi-task models, applying either masking only (MT-mask) or both masking and task-weighting (MT-mask-weight). The loss function for multi-label classifier utilizes sigmoid cross-entropy and was averaged for each batch as described below.

Loss function for multi-label classifier using masking:

Loss = Σ_i,t_ m_i,t_ ∗ [y_i,t_ ∗ −log(sigmoid(x_i,t_)) + (1−y_i,t_) ∗ −log(1−sigmoid(x_i,t_))]/Σ_i,t_ m_i,t_ (1)

Loss function for multi-label classifier using masking and task-weighting:

Loss = Σ_i,t_ m_i,t_ ∗ w_t_ ∗ [y_i,t_ ∗ −log(sigmoid(x_i,t_)) + (1−y_i,t_) ∗ ‒log(1−sigmoid(x_i,t_))]/Σ_i,t_ m_i,t_ ∗ w_t_ (2)

where x_i,t_ stands for the predicted value for the binding activity between a query compound i and target t. y_i,t_ indicates the true activity corresponding to x_i,t_ with 1 for positive and 0 for negative. m_i,t_ is an element representing a mask with 1 for a known interaction and 0 for an unknown. w_t_ is the weight of a target t which is the reversal of the number of known activities of the target.

Meanwhile, the multi-task architecture using multiple binary classifiers (MT-binary) is free from many issues derived from batch generation. This is because they can build independent mini-batches for each task [2,23]. Nonetheless, high computation cost in both time and memory is problematic for large-scale targets. In this study, we sequentially trained each task with equally sized mini-batches composed of known activities alone. If the number of known activities is smaller than the batch size, known activities are duplicated until the size is matched. In addition, early stopping [33] is applied for each task to avoid over-training. When a task starts to be over-fitted, the model is saved and validated for the task. Multi-task learning for the remaining targets then continues until all tasks are complete. Single-task learning also adopts this mechanism; however, it does not share any common layers between tasks (Figure 1b). The loss function for binary classifier utilizes softmax cross-entropy and average for each batch.

Loss function for multiple binary classifier defined for each target:

Loss = Σ_i_ [y_i_ ∗ −log(exp(x_i,0_)/(exp(x_i,0_) + exp(x_i,1_))) + (1-y_i_) ∗ −log(exp(x_i,1_)/(exp(x_i,0_) + exp(x_i,1_)))]/N (3)

where x_i,0_ and x_i,1_ represents the predicted value for binding and non-binding activities of a compound i, respectively. y_i_ indicates the true activity between compound i and the target with 1 for positive and 0 for negative. N indicates the number of compounds in a batch.

TensorFlow (version 1.10.1) [34] was used to implement the architectures described above. For both multi-task learning and single-task learning, only a single model is built in the workspace to reduce time and memory for building novel models. Switching tasks and sharing of common layers are controlled by the TensorFlow initializer and saver, which is appropriate for large-scale multi-task learning.

### 2.4. Nested Cluster Cross Validation and Target-AUC

According to a study by Mayr et al., machine learning prediction for the chemical database has a variety of potential biases [22]. Firstly, compound series bias easily occurs because databases have highly similar compounds that share common scaffolds [25]. Hence, a prediction model that is optimized for a specific scaffold may overestimate the performance, which is not suitable for activity prediction of novel drugs. Secondly, hyperparameter selection that is optimized for a specific algorithm is also problematic because it does not allow a fair comparison between methods. To overcome such biases, we adopted nested cluster cross validation for comparison between various DNNs [35]. Details of the nested cluster cross validation is described in Supplementary File S-2.

ROC-AUC is one of the most common metrics to evaluate the performance of binary classification (active or inactive). However, based on the purpose of multi-task learning, validating the ROC curve across all targets cannot represent the prediction accuracy for individual targets. Some of the tasks may suffer from low prediction power while other tasks may display overwhelming accuracy. To check such skewed performance, we used target-AUC, which indicates the average ROC-AUC over validation fold for each task [22]. Validating the mean and variance of target-AUC across targets more precisely represents the performance of activity prediction for the respective targets. Moreover, the robustness of multi-task architectures can be evaluated by measuring how many tasks outperform single-task learning [23].

### 2.5. Target Clustering Based on Sequence Similarities at Binding Domains

As high inter-task correlation is known to improve the effectiveness of multi-task learning [23], we speculated that human targets with high similarities in their binding domains can be better trained using multi-task learning. More than 70% of human targets in the ChEMBL database have sequences of known binding domains (774 among 1067 human targets). Targets were clustered by their binding domain similarities using normalized BLOSUM62 (Blocks of Amino acid substitution matrix) distance [36]. Distance between two sequences was calculated using Equation (4) below. Subsequently, the lowest distance was selected as the distance between two targets if there were multiple binding domains for a target. The “pairwise2” function in the Biopython [37] package was used for the distance calculation and hierarchical clustering was done using fastcluster [38] linkage with the “average” option. To determine the best threshold for similarity clustering from the linkage, various flat clusters of targets were made for different distance cutoffs, ranging from 0.2 to 0.4.

Normalized BLOSUM62 distance between two protein sequences:

Normalized BLOSUM62 distance = (score1−pair_score) ∗ (score2-pair_score)/(score1 ∗ score2)(4)

where score1 and score2 indicate the BLOSUM62 alignment scores between the same sequences for each sequence. The pair_score is defined as the local alignment score between two sequences using the BLOSUM62 matrix with a gap open penalty of ‒1 and a gap extension penalty of −0.1.

### 2.6. Multiple Partial Multi-Task Deep Neural Network (MPMT-DNN)

We developed a novel multi-task learning strategy that is effective for molecular binding prediction of human targets in ChEMBL. This was derived with reference to the results from various validations and comparisons of DNNs. Instead of performing multi-task learning for all human targets at once, we clustered human targets based on their sequence similarities in their binding domain sequences. For each cluster, targets in a cluster were trained using multi-task learning (Figure 1c). Targets without binding domain sequences or neighboring targets were trained by single-task learning. The same standard of validation was applied to the novel algorithm for comparison to other DNNs. The data and python codes that were used to implement the pipeline can be downloaded at GitHub: https://github.com/KyoungYeulLee/MPMT.

## 3. Results

### 3.1. Workflow for Deep Neural Networks Evaluation

**Figure 2:**
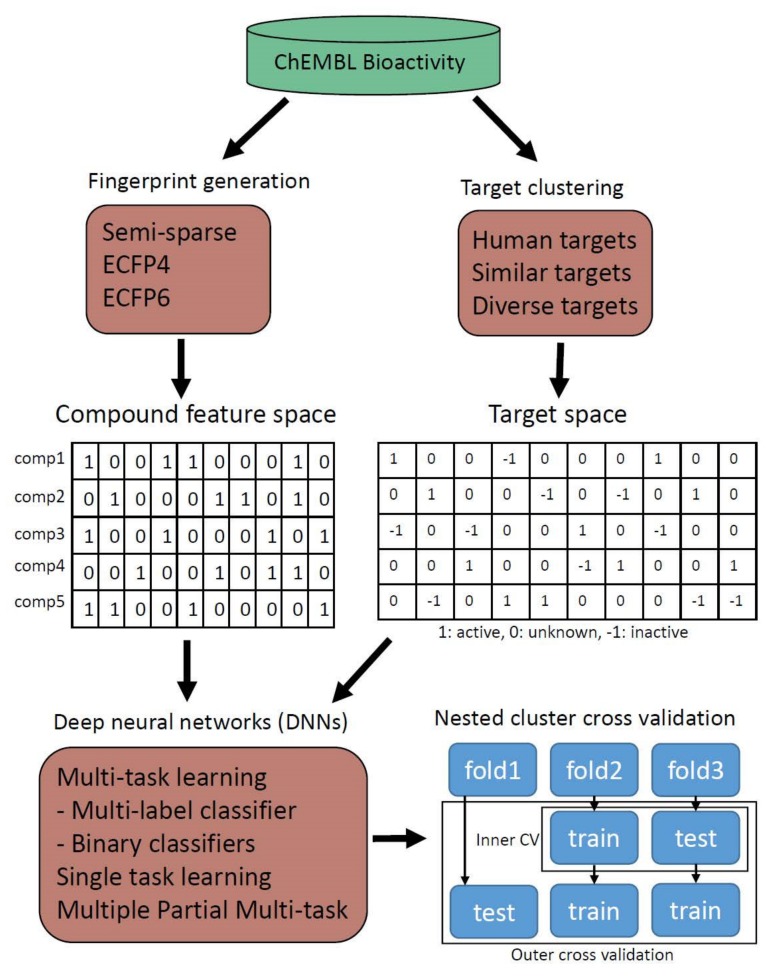
Overall workflow scheme. Compound feature data and target activity data were curated from the ChEMBL database. Various deep neural network architectures including multi-task and single-task learning were trained and tested by nested cluster cross validation.

Bioactivity data from ChEMBL (version 23) was divided into two types of python array data. Feature space for binding compounds was generated for Semi-sparse, ECFP4, and ECFP6 fingerprints. Target space representing activities between compounds and protein targets was also generated. All possible activity types including active interactions, inactive interactions, and unknown interactions were distinguished by imposing different values. Targets were also clustered based on their sequence similarities at known binding domains to build data with different target compositions. Various deep neural architectures including multi-task and single-task learning were Constructed by TensorFlow, which were trained and tested with bioactivity data. Each DNN was tested fairly and compared by nested cluster cross validation, which is constructed to avoid overfitting to specific compound scaffolds or hyperparameters. Using this normalized scheme, various multi-task strategies were evaluated to find the best practices for binding prediction of human targets (Figure 2).

### 3.2. Data Composition and Target Clusters

We collected targets that are only expressed in Homo sapiens (tax id = 9606). Targets with at least one active and one inactive data for each fold in compound clusters (see Supplementary File S-2) were gathered to perform a ROC-AUC calculation for each target in cross-validation. In the ChEMBL database, the number of human targets that met the standard was 1067. Targets were clustered by sequence similarities at binding domains with different distance cutoffs of 0.2, 0.3, and 0.4 (see materials and methods) (Table 1). From the clusters, we selected target sets with various target compositions. Firstly, we built target sets composed of similar targets for each distance cutoff (Table 2). For example, similar targets with d ≤ 0.3 indicates a target set where targets are similar to each other by a maximum distance of 0.3. For simplicity, the largest group of targets for each distance was selected as the similar target set. We also defined a diverse target set, which contained dissimilar targets by a distance of at least 0.4. Compounds were also selected for each target set as they had at least one interaction with the targets. The resulting data composition is presented in detail in Table 2.

### 3.3. Performance Validation of Deep Neural Architectures

Target-AUC, which indicates an average ROC-AUC over validation fold for each target, was used as a standard to quantitatively compare the performance of deep neural architectures. As target-AUC can measure prediction performance for each target, we could precisely verify the strengths and weaknesses of various architectures and find practical strategies for different target compositions. Not only average target-AUC across targets, but also standard deviation, maximum, and minimum of the target-AUC were measured, providing insights on the unique properties of each architecture. In addition, the robustness of multi-task architectures, representing the ability of networks to improve performance across all targets, was examined using single-task learning as a baseline [23]. The proportion of targets where the prediction outperformed single-task learning was measured as a standard for robustness. The overall target-AUCs for various combinations between architectures and target sets are shown in Table 3. The table contains mean target-AUC across targets and the best performance is highlighted for each target set.

The result clearly shows that multi-task learning can outperform single-task learning when targets are similar. However, for diverse target sets or overall human targets, no multi-task architecture could outperform single-task learning. Although it is known that inter-task correlation is an important factor for the performance of multi-task learning, little is known about the adverse effects of multi-task learning. This result suggests that multi-task learning may reduce prediction performance when the target list is not cautiously selected. Furthermore, simply applying multi-task architectures across human targets can be problematic. Nevertheless, multi-task architecture demonstrated its effectiveness when applied to targets with high similarity. The details of several meaningful cases were further examined and the exact difference between deep-neural architectures was compared to elucidate their properties and the best practices. The performance using ECFP4 and ECFP6 as compound representation is reported in Supplementary File S-3, which enables similar conclusions as described above.

### 3.4. Case Study for Specific Target Sets

#### 3.4.1. Human Targets

**Figure 3:**
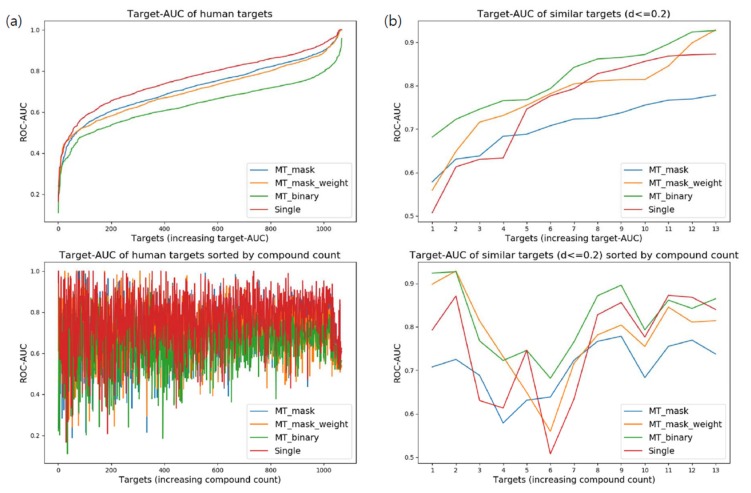
Comparison of target-area under curve (AUC) distribution for various deep neural architectures: (**a**) Target-AUC distribution of networks trained for overall human targets. Targets of the upper graph were independently sorted by the order of increasing target-AUC for each architecture. Targets of the lower graph were arranged by the number of associated compounds of each target (targets were sorted by the same order across all architectures); (**b**) Target-AUC distribution of networks trained for similar human targets (d ≤ 0.2).

When testing overall human targets without considering similarity, the prediction performance of single-task learning outperformed that of multi-task learning. Surprisingly, target-AUC for the single-task network generally showed better performance across targets (Figure 3a). More than 70% of targets displayed their best performance when trained by single-task learning. However, the low minimum target-AUC for single-task learning remained a defect. The failure of multi-task learning might be caused by insufficient training for the difference between dissimilar targets. As multi-task networks share hidden layers across targets, the difference between targets should be trained by target-specific layers. This result implies the importance of target selection for fine multi-task learning (Table 4).

#### 3.4.2. Similar Targets (d ≤ 0.2)

Multi-task network with binary classifiers (MT-binary) showed the most qualified performance when validating highly similar targets (d ≤ 0.2). Of the targets, 76.9% had better prediction performance using MT-binary than single-task learning (Figure 3b). An interesting aspect of this result is the low performance of MT-mask. We interpreted this phenomenon as an amplification of data imbalance between targets. As a similar target set (d ≤ 0.2) has only 13 targets in total, common layers of MT-mask can be overfitted more easily to the target with large volumes of data. MT-mask-weight and MT-binary can keep balance between tasks during training. Moreover, MT-binary can generate mini-batches for targets independently, which naturally enables strict balancing between targets. Such stability of MT-binary to data imbalance would serve as a reason to adopt binary classifiers for cases with a relatively small target set (Table 5).

### 3.5. Multiple Partial Multi-Task Learning

Because multi-task learning is promising for similar targets but inaccurate for diverse targets, a more sophisticated technique is needed for large-scale human drug targets. We developed a technique that takes advantage of both multi-task learning and single-task learning. Multi-task learning has the strength of using information from targets with qualified data to train targets with low accuracy. Single-task learning can more clearly identify the difference between targets than multi-task learning. In this technique, multi-task learning (MT-binary) is performed multiple times for partial target sets by clustering similar human targets. Through this process, diverse targets did not share common layers, thereby enabling the identification of the differences between dissimilar targets. For similar targets, multi-task learning improved the accuracy, especially for the targets with low performance by single-task learning. We named this technique Multiple Partial Multi-task learning (MPMT).

**Figure 4:**
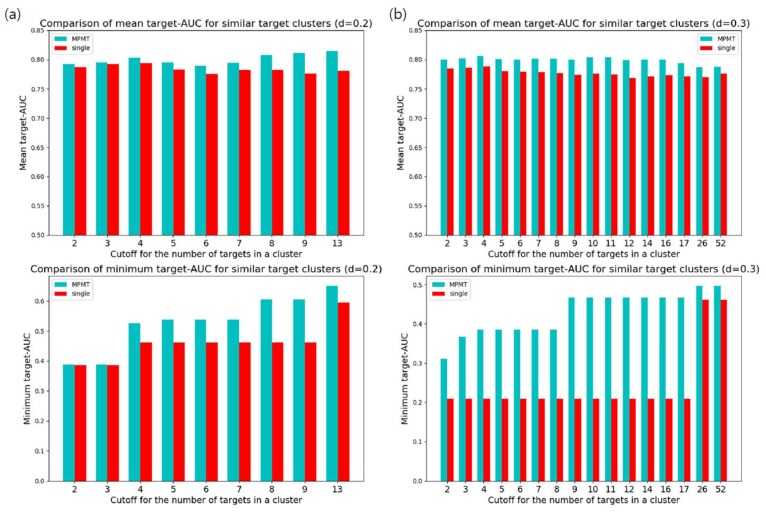
Comparison of target-area under curve (AUC) between multiple partial multi-task learning and single-task learning for similar target clusters. Clusters containing more targets than the cutoff were selected and the target-AUC of the targets in the clusters were evaluated: (**a**) Comparison of target-AUC for similar targets clustered with a distance of 0.2. The upper bar graph represents the average target-AUC with different cutoffs for the number of targets in a cluster. Below represents the minimum target-AUC within selected clusters; (**b**) Comparison of target-AUC for similar targets clustered with a distance of 0.3.

The performance of MPMT must be compared to that of single-task learning. First, we compared target-AUC for targets included in the similar target clusters. As a sufficient number of targets for effective multi-task learning were ambiguous, we selected clusters using different cutoffs for the minimal number of targets in a cluster. For selected clusters, MPMT trained targets by multi-task learning for each cluster. The overall targets in selected clusters were also trained by single-task learning for comparison. Clustering of targets used normalized BLOSUM62 distance with a distance cutoff of 0.2 or 0.3. Mean and minimum target-AUC were compared between MPMT and single-task learning for various cutoffs and clustering distances (Figure 4). The result clearly shows that MPMT generally outperformed single-task learning, especially for targets clustered by a distance of 0.3.

**Figure 5:**
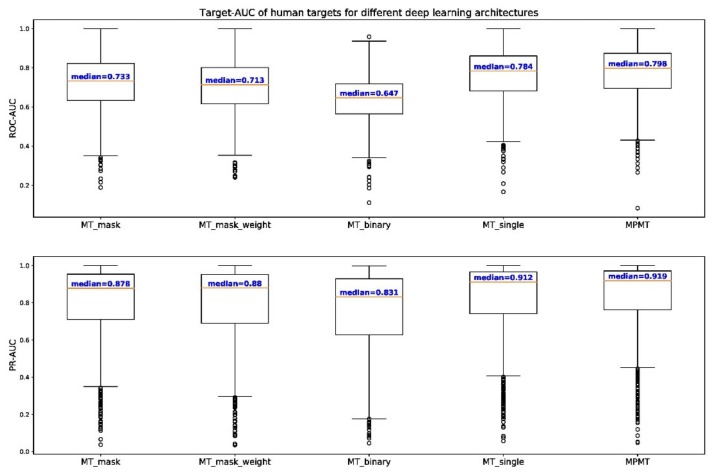
Box plot of target-AUC of different deep learning architectures for 1067 human targets. The upper graph shows the distribution of target-AUC for ROC while the graph below shows the distribution for precision-recall (PR) curve indicated as PR-AUC. The median target-AUC is represented as a red line and outliers outside the fences (1.5 times the interquartile range) are indicated as small circles.

We also compared the performance of MPMT to that of other deep neural architectures for 1067 human targets (Table 6). Human targets were clustered by the distance cutoff of 0.3. Each cluster with at least 2 targets was trained by MT-binary to retrieve the benefits of multi-task learning for as many targets as possible. Targets that did not cluster with any other targets were subjected to single-task learning. MPMT outperforms both multi-task and single-task learning with 65.1% of targets predicted more accurately compared to baseline. To compare the performances for different architectures visually, box plots of target-AUC for human targets were drawn (Figure 5). For not only ROC but also the Precision-Recall (PR) curve, target-AUC for MPMT had the highest median among them all. For the rank-sum test, p-value equals 3.34 × 10^−3^ for the alternative hypothesis that MPMT shows higher target-AUC compared to single-task learning, and p-values are even smaller when compared to other architectures. This result concludes that the deep learning model that is built by MPMT shows the most reliable binding prediction for human targets.

### 3.6. Utilizing MPMT for Drug Discovery

#### 3.6.1. Performance Improvement for Inaccurate Target Models

Some of the human targets cannot be trained properly because of the lack of known active compounds, biased compound structures, or network overtraining. As multi-task learning is known to relieve such bias, MPMT was applied for the targets with low accuracy when trained by single-task learning. Targets with ROC-AUC ranging from 0.5 to 0.6 for single-task learning were trained by MPMT with a distance cutoff of 0.2 and the performances were compared. The results clearly showed that the prediction accuracy of the target models improved when they were trained by MPMT. The rate of improvement was about 84% as the performances of 16 targets out of 19 targets were improved. Some of the targets improved greatly such as Tyrosine-protein kinase Lyn (ChEMBL ID: 3905) of which ROC-AUC increased from 0.574 to 0.758 using MPMT. The performance of two targets were slightly decreased and only one target, Peptidyl-prolyl cis-trans isomerase FKBP5 (ChEMBL ID: 2052031), showed a significant performance decrease from 0.5921 to 0.4878. Such decreases might be caused by selecting the wrong binding domains during target clustering or underestimating the difference of the target from similar targets during multi-task learning.

#### 3.6.2. Hit Finding for a Specific Human Target

Even though the performance of MPMT seems better than that of single-task learning statistically, we need to check whether it is helpful for finding potential drug candidates. For this purpose, we investigated approved drugs binding to Tyrosine-protein kinase Lyn and assessed the probability of MPMT to identify those drugs as potential candidates. The results compared with single-task learning are represented in Table 7. It shows that MPMT can estimate those ligands that are more promising for drug candidates compared to single-task learning. Using MPMT, researchers can select promising drug candidates for a specific target fast and precisely. However, considerably inferior ranks for the drug target can be problematic to find novel targets for drugs or to predict off-target effects accurately. The models would be further improved if they were capable of multiple target prediction of ligands in the future.

## 4. Discussion

Although the importance of inter-task correlation when performing multi-task learning is known, the adverse effect of multi-task learning for dissimilar tasks has not been adequately investigated. In this study, we found that multi-task learning can interfere with proper target prediction if the degree of similarity between the targets is low. By applying multi-task learning to large-scale targets, such as overall human targets, significantly worse performance was achieved compared to that with single-task learning. This suggests that the target set for multi-task learning should be selected more carefully for practical purposes (i.e., a virtual screening of novel drugs for general human targets).

According to the results of this study, clustering human targets based on their similarity may be effective in improving the accuracy of molecular binding prediction for large-scale human targets. In this study, target similarity was calculated using sequence similarity at binding domains. This improved the efficiency of multi-task learning for similar human targets. In addition, through the strategy termed MPMT, target sets with high similarity were trained by multi-task learning while other targets were trained by single-task learning. The results demonstrate that, compared to previous methods, the prediction performance for overall human targets in ChEMBL was improved. 

However, a considerable number of targets remain unclustered because of unknown binding domain or low similarity to other targets, thereby functioning as an obstacle of MPMT in achieving significant improvement in prediction accuracy. If we can cluster targets more precisely to ensure most targets have neighboring targets, a further improvement in the performance of MPMT is expected. The novel clustering method may exploit the characteristics of known drugs or the family of targets besides sequence similarity. In addition, the algorithm for partial multi-task learning can be developed beyond a simple divide and conquer method. Therefore, MPMT is expected to be a technique that will more likely improve in the future.

Increasing the performance of drug-binding predictions through multi-task learning can increase the efficiency of drug development. Accurate in-silico binding predictions will dramatically reduce the time and cost that is necessary for drug screening [39]. Additionally, the effectiveness and off-target effect of novel drugs can be predicted beforehand to reduce the failure rate of clinical trials. In addition, reliable DNNs for molecular binding prediction would help to improve the performance of deep learning-based drug designs. Recent advancements in reinforced learning techniques [40] enable predictive models to improve the performance of generation models [41]. In particular, a deep learning-based molecular binding predictor is expected to be flexibly combined with a compound generation model to help design novel compounds with desirable binding activities to specific targets.

## Figures and Tables

**Table 1:** The specification of target clusters with distance cutoff (annotated as “d”).

	Cluster (d = 0.2)	Cluster (d = 0.3)	Cluster (d = 0.4)
Distance cutoff ^1^	0.2	0.3	0.4
Maximum size ^2^	13	52	106
The number of clusters ^3^	152	132	75
The total number of targets ^4^	506	608	696

^1^ Normalized BLOSUM62 distance. ^2^ The number of targets in the largest cluster. ^3^ The number of clusters with at least 2 targets. ^4^ The total number of targets included in clusters with at least 2 targets.

**Table 2:** Data composition of various target sets divided by taxonomy and target clustering.

Type	Human Targets	Similar Targets (d ≤ 0.4) ^1^	Similar Targets (d ≤ 0.3)	Similar Targets (d ≤ 0.2)	Diverse Targets (d > 0.4) ^2^
Targets	1067	106	52	13	120
Compounds	608,160	47,432	30,956	5154	73,515

^1^ Distances between the targets are at most 0.4. ^2^ Distances between the targets are at least 0.4.

**Table 3:** Average target area under curve for different architectures and target sets.

Target Set:	Similar Targets (d ≤ 0.4)	Similar Targets (d ≤ 0.3)	Similar Targets (d ≤ 0.2)	Diverse Targets (d > 0.4)	Human Targets
MT-mask ^1^	**0.802**	**0.805**	0.706	0.733	0.718
MT-mask-weight ^2^	0.746	0.777	0.777	0.747	0.706
MT-binary ^3^	0.787	0.789	**0.820**	0.729	0.636
Single-task	0.789	0.776	0.757	**0.79**	**0.762**
Num targets ^4^	106	52	13	120	1067

^1^ Multi-task learning using multi-label classifier with masking. ^2^ Multi-task learning using multi-label classifier with masking and task-weighting. ^3^ Multi-task learning using multiple binary classifiers. ^4^ The number of targets within a target set.

**Table 4:** Comparison of deep neural architectures for human targets.

Architecture	MT-Mask	MT-Mask-Weight	MT-Binary	Single-Task
Mean AUC	0.718	0.706	0.636	**0.762**
Std AUC	0.137	0.132	0.117	0.133
Max AUC	1.000	1.000	0.959	1.000
Min AUC	0.189	**0.240**	0.111	0.167
Robustness	294/1067 (27.6%)	257/1067 (24.1%)	113/1067 (10.6%)	**Baseline**

**Table 5:** Comparison of deep neural architectures for a similar target set (d ≤ 0.2).

Architecture	MT-Mask	MT-Mask-Weight	MT-Binary	Single-Task
Mean AUC	0.706	0.777	**0.820**	0.757
Std AUC	0.058	0.095	0.076	0.116
Max AUC	0.778	0.928	0.927	0.872
Min AUC	0.579	0.560	**0.682**	0.508
Robustness	3/13 (23.1%)	6/13 (46.2%)	**10/13 (76.9%)**	Baseline

**Table 6:** Comparison of deep neural architectures for human targets including Multiple Partial Multi-Task (MPMT).

Architecture	MT-Mask	MT-Mask-Weight	MT-Binary	Single-Task	MPMT
Mean AUC	0.718	0.706	0.636	0.762	**0.776**
Std AUC	0.137	0.132	0.117	0.133	0.132
Max AUC	1.000	1.000	0.959	1.000	1.000
Min AUC	0.189	**0.240**	0.111	0.167	0.083
Robustness	27.6%	24.1%	10.6%	Baseline	**65.1%**

**Table 7:** Binding prediction of approved drugs of Tyrosine-protein kinase Lyn.

Architectures	Multiple Partial Multi-Task		Single-Task	
Approved Drugs	Score	Rank ^1^	Score	Rank
dasatinib	0.9123	277	0.4545	381
bosutinib	0.9999	83	0.9989	162
vandetanib	1.0000	54	0.9774	244
nilotinib	0.9999	75	0.9621	262
sorafenib	0.9976	151	0.9965	192

^1^ Rank for the probability of Tyrosine-protein kinase Lyn to be a target protein among 506 human targets.

## Supplementary Materials

The following are available online at https://www.mdpi.com/2073-4425/10/11/906/s1, Supplementary S-1: SQL script for data curation from the ChEMBL database, Supplementary S-2: Details about nested cluster cross validation, Supplementary S-3: Comparison of deep neural architectures using ECFP as compound representation. Deposit for data and software source code: https://github.com/KyoungYeulLee/MPMT.

## References

[1] Wallach I., Dzamba M., Heifets A. (2015). AtomNet: A Deep Convolutional Neural Network for Bioactivity Prediction in Structure-based Drug Discovery. arXiv.

[2] Ramsundar B., Kearnes S., Riley P., Webster D., Konerding D., Pande V. (2015). Massively Multitask Networks for Drug Discovery. arXiv.

[3] Kola I., Landis J. (2004). Can the pharmaceutical industry reduce attrition rates?. Nat. Rev. Drug Discov..

[4] Thomas D., Burns J., Audette J., Carroll A., Dow-Hygelund C., Hay M. (2016). Clinical Development Success Rates. BioMedTracker.

[5] Vanhaelen Q., Mamoshina P., Aliper A.M., Artemov A., Lezhnina K., Ozerov I., Labat I., Zhavoronkov A. (2017). Design of efficient computational workflows for in silico drug repurposing. Drug Discov. Today.

[6] Kitchen D.B., Decornez H., Furr J.R., Bajorath J. (2004). Docking and scoring in virtual screening for drug discovery: Methods and applications. Nat. Rev. Drug Discov..

[7] Wang L., Wu Y., Deng Y., Kim B., Pierce L., Krilov G., Lupyan D., Robinson S., Dahlgren M.K., Greenwood J. (2015). Accurate and reliable prediction of relative ligand binding potency in prospective drug discovery by way of a modern free-energy calculation protocol and force field. J. Am. Chem. Soc..

[8] Ma J., Sheridan R.P., Liaw A., Dahl G.E., Svetnik V. (2015). Deep neural nets as a method for quantitative structure-activity relationships. J. Chem. Inf. Model..

[9] Dahl G., Jaitly N., Salakhutdinov R. (2014). Multi-task Neural Networks for QSAR Predictions. arXiv.

[10] Jarrett K., Kavukcuoglu K., Ranzato M., LeCun Y. What is the best multi-stage architecture for object recognition?. Proceedings of the 2009 IEEE 12th International Conference on Computer Vision.

[11] Schmidhuber J. (2015). Deep Learning in neural networks: An overview. Neural Netw..

[12] Walters W.P., Murcko M.A., Ajay (1998). Can we learn to distinguish between “drug-like” and “nondrug-like” molecules?. J. Med. Chem..

[13] Burden F.R., Ford M.G., Whitley D.C., Winkler D.A. (2000). Use of Automatic Relevance Determination in QSAR Studies Using Bayesian Neural Networks. J. Chem. Inf. Comput. Sci..

[14] Svetnik V., Liaw A., Tong C., Christopher Culberson J., Sheridan R.P., Feuston B.P. (2003). Random Forest: A Classification and Regression Tool for Compound Classification and QSAR Modeling. J. Chem. Inf. Comput. Sci..

[15] Du H., Wang J., Hu Z., Yao X., Zhang X. (2008). Prediction of fungicidal activities of rice blast disease based on least-squares support vector machines and project pursuit regression. J. Agric. Food Chem..

[16] Krizhevsky A., Sutskever I., Hinton G.E. (2006). ImageNet Classification with Deep Convolutional Neural Networks Alex. Advances in Neural Information Processing Systems.

[17] (2016). AI Research Deep Neural Networks for Acoustic Modeling in Speech Recognition—AI Research. Http://Airesearch.Com.

[18] Collobert R., Weston J. (2008). A unified Architecture for Natural Language Processing: Deep Neural Networks with Multitask Learning. ICML.

[19] Gaulton A., Hersey A., Nowotka M.L., Bento A.P., Chambers J., Mendez D., Mutowo P., Atkinson F., Bellis L.J., Cibrian-Uhalte E. (2017). The ChEMBL database in 2017. Nucleic Acids Res..

[20] Unterthiner T., Mayr A., Klambauer G., Steijaert M., Wegner J.K., Ceulemans H. Deep Learning as an Opportunity in Virtual Screening. Proceedings of the Deep Learning Workshop at NIPS.

[21] Keiser M.J., Roth B.L., Armbruster B.N., Ernsberger P., Irwin J.J., Shoichet B.K. (2007). Relating protein pharmacology by ligand chemistry. Nat. Biotechnol..

[22] Mayr A., Klambauer G., Unterthiner T., Steijaert M., Wegner J.K., Ceulemans H., Clevert D.A., Hochreiter S. (2018). Large-scale comparison of machine learning methods for drug target prediction on ChEMBL. Chem. Sci..

[23] Ramsundar B., Liu B., Wu Z., Verras A., Tudor M., Sheridan R.P., Pande V. (2017). Is Multitask Deep Learning Practical for Pharma?. J. Chem. Inf. Model..

[24] Groom C.R., Hopkins A.L. (2002). The druggable genome. Nat. Rev. Drug Discov..

[25] Sheridan R.P. (2013). Time-split cross-validation as a method for estimating the goodness of prospective prediction. J. Chem. Inf. Model..

[26] Unterthiner T., Mayr A., Klambauer G., Hochreiter S. (2015). Toxicity Prediction using Deep Learning. arXiv.

[27] Davies M., Nowotka M., Papadatos G., Dedman N., Gaulton A., Atkinson F., Bellis L., Overington J.P. (2015). ChEMBL web services: Streamlining access to drug discovery data and utilities. Nucleic Acids Res..

[28] Michael W., Axmark D., DuBois P. (2002). Mysql Reference Manual.

[29] Weininger D. (1988). SMILES, a Chemical Language and Information System: 1: Introduction to Methodology and Encoding Rules. J. Chem. Inf. Comput. Sci..

[30] Standardizer (J. Chem. Version 16.4.4) Developed by ChemAxon. https://chemaxon.com/products/chemical-structure-representation-toolkit.

[31] Rogers D., Hahn M. (2010). Extended-Connectivity Fingerprints. J. Chem. Inf. Mod..

[32] Landrum G. RDKit: Open-Source Cheminformatics. http://www.rdkit.org.

[33] Bengio Y., Louradour J., Collobert R., Weston J. (2009). Curriculum learning. Journal of the American Podiatry Association.

[34] Abadi M., Agarwal A., Paul Barham E.B., Chen Z., Citro C., Greg S., Corrado A.D., Dean J., Devin M., Sanjay Ghemawat I.G. (1983). TensorFlow: Large-scale machine learning on heterogeneous systems. Methods Enzymol..

[35] Baumann D., Baumann K. (2014). Reliable estimation of prediction errors for QSAR models under model uncertainty using double cross-validation. J. Cheminform..

[36] Song D., Chen J., Chen G., Li N., Li J., Fan J., Bu D., Li S.C. (2015). Parameterized BLOSUM matrices for protein alignment. IEEE/ACM Trans. Comput. Biol. Bioinforma..

[37] Cock P.J.A., Antao T., Chang J.T., Chapman B.A., Cox C.J., Dalke A., Friedberg I., Hamelryck T., Kauff F., Wilczynski B. (2009). Biopython: Freely available Python tools for computational molecular biology and bioinformatics. Bioinformatics.

[38] Müllner D. (2015). fastcluster: Fast Hierarchical, Agglomerative Clustering Routines for R and Python. J. Stat. Softw..

[39] Shoichet B.K. (2006). Virtual screening of chemical libraries. HHS Author Manuscr..

[40] Guimaraes G.L., Sanchez-Lengeling B., Farias P.L.C., Aspuru-Guzik A. (2017). Objective-Reinforced Generative Adversarial Networks (ORGAN) for Sequence Generation Models. arXiv.

[41] De Cao N., Kipf T. (2018). MolGAN: An implicit generative model for small molecular graphs. arXiv.

