# Supplementary material for: In-Silico Molecular Binding Prediction for Human Drug Targets Using Deep Neural Multi-Task Learning
Source: Genes (Basel). 2019 Nov 7;10(11):906. doi: 10.3390/genes10110906 (PMC6896155; doi:10.3390/genes10110906)
Supplement: Supplementary file 1 [file genes-10-00906-s001.zip › genes-597313-supplementary/Supplementary_S-2.docx]

**Details about nested cluster cross validation**

Nested cluster cross validation is composed of two concepts: 1) cluster cross-validation [1] and 2) nested cross-validation [2]. The cluster cross validation is the idea that compounds are clustered based on their structure similarities and the clusters are distributed into cross-validation folds. By this means, any of the compounds in the test set do not share the scaffolds with the compounds in training set, which is more realistic in development of novel drugs [3]. In this study, ECFP4 features were used to represent structures of compounds and distances between compounds were calculated applying jaccard distance. Instead of using hierarchical clustering in Scipy module, we made use of Fastcluster module [4] to cluster the large scale compounds (794,950 compounds). “single linkage” option was applied for clustering to make sure that any compounds in different clusters have dissimilar scaffolds. In this study, compounds with jaccard distance higher than 0.3 were separated into different clusters. For cross-validation, we built 3 structurally distinct compound sets by grouping the clusters randomly (about 264,983 compounds for each fold).

Nested cross-validation is necessary to select the best hyperparameter optimized for each deep neural architectures. If we arbitrary select and apply the same hyperparameter across all the architectures, the architecture suitable for the specific hyperparameter may get better performance regardless of its rationality. Therefore, hyperparameter needs to be optimized for each architecture independently for fair comparison. However, using test set to optimize hyperparameter is forbidden in cross-validation. In nested cross-validation, training set is further divided into inner training set and inner test set for inner cross-validation. Various hyperparameters are tested in inner cross-validation and the hyperparameter having the best performance is selected for outer cross-validation. Inner cross-validation is repeatedly performed for different outer fold so that the principle of cross-validation can remain not violated. The hyperparameter grid used for inner cross-validation and the code for nested cross-validation can be obtained at https://github.com/KyoungYeulLee/MPMT.

1. Sheridan, R.P. Time-split cross-validation as a method for estimating the goodness of prospective prediction. *J. Chem. Inf. Model.* **2013**, *53*, 783–790.

2. Baumann, D.; Baumann, K. Reliable estimation of prediction errors for QSAR models under model uncertainty using double cross-validation. *J. Cheminform.* **2014**, *6*, 1–19.

3. Mayr, A.; Klambauer, G.; Unterthiner, T.; Steijaert, M.; Wegner, J.K.; Ceulemans, H.; Clevert, D.A.; Hochreiter, S. Large-scale comparison of machine learning methods for drug target prediction on ChEMBL. *Chem. Sci.* **2018**, *9*, 5441–5451.

4. Müllner, D. fastcluster : Fast Hierarchical, Agglomerative Clustering Routines for R and Python . *J. Stat. Softw.* **2015**, *53*.
