# Supplementary material for: In-Silico Molecular Binding Prediction for Human Drug Targets Using Deep Neural Multi-Task Learning
Source: Genes (Basel). 2019 Nov 7;10(11):906. doi: 10.3390/genes10110906 (PMC6896155; doi:10.3390/genes10110906)
Supplement: Supplementary file 1 [file genes-10-00906-s001.zip › genes-597313-supplementary/Supplementary_S-3.docx]

**Comparison of deep neural architectures using ECFP as compound representation**

Average target-AUC across various deep neural architectures and target sets were validated for compound data using ECFP as features. The resulting tables S1 (ECFP4) and table S2 (ECFP6) confirm the fact that multi-task learning can outperform single-task learning when targets are similar while single-task learning works better for diverse target or overall human targets. The specific multi-task architecture outstanding for each target set is different with the result of semi-sparse features (Table 3). Especially MT-binary for similar targets seems to be less effective when using ECFP as a compound feature. As partial multi-task learning can be implemented using MT-binary more efficiently, semi-sparse feature would be practical for MPMT architecture.

**Table S1.** Average target AUC for different architectures and target sets (ECFP4).

| **Target set:** | Similar targets (d≤0.4) | Similar targets (d≤0.3) | Similar targets (d≤0.2) | Diverse targets (d>0.4) | Human targets |
| --- | --- | --- | --- | --- | --- |
| MT-mask ^1^ | **0.828** | 0.779 | 0.758 | 0.769 | 0.702 |
| MT-mask-weight ^2^ | 0.814 | 0.765 | **0.818** | 0.779 | 0.654 |
| MT-binary ^3^ | 0.789 | **0.802** | 0.782 | 0.774 | 0.664 |
| Single-task | 0.809 | 0.797 | 0.776 | **0.799** | **0.784** |
| Num targets ^4^ | 106 | 52 | 13 | 120 | 1067 |

**Table S2.** Average target AUC for different architectures and target sets (ECFP6).

| **Target set:** | Similar targets (d≤0.4) | Similar targets (d≤0.3) | Similar targets (d≤0.2) | Diverse targets (d>0.4) | Human targets |
| --- | --- | --- | --- | --- | --- |
| MT-mask ^1^ | **0.806** | 0.812 | **0.811** | 0.752 | 0.691 |
| MT-mask-weight ^2^ | 0.792 | **0.826** | 0.787 | 0.765 | 0.665 |
| MT-binary ^3^ | 0.780 | 0.816 | 0.771 | 0.77 | 0.657 |
| Single-task | 0.800 | 0.765 | 0.794 | **0.793** | **0.778** |
| Num targets ^4^ | 106 | 52 | 13 | 120 | 1067 |
